# Cygnet River Virus, a Novel Orthomyxovirus from Ducks, Australia

**DOI:** 10.3201/eid1812.120500

**Published:** 2012-12

**Authors:** Allan Kessell, Alex Hyatt, Debra Lehmann, Songhua Shan, Sandra Crameri, Clare Holmes, Glenn Marsh, Catherine Williams, Mary Tachedjian, Meng Yu, John Bingham, Jean Payne, Sue Lowther, Jianning Wang, Lin-Fa Wang, Ina Smith

**Affiliations:** Author affiliations: Gribbles Pathology, Glenside, South Australia, Australia (A. Kessell);; Commonwealth Scientific and Industrial Research Organization, Geelong, Victoria, Australia (A. Hyatt, S. Shan, S. Crameri, C. Holmes, G. Marsh, C. Williams, M. Tachedjian, M. Yu, J. Bingham, J. Payne, S. Lowther, J. Wang, L.-F. Wang, I. Smith);; Kangaroo Island Veterinary Clinic, Kangaroo Island, South Australia, Australia (D. Lehmann)

**Keywords:** Cygnet River virus, orthomyxovirus, ducks, Muscovy ducks, Cairina moschate, Australia, viruses

## Abstract

A novel virus, designated Cygnet River virus (CyRV), was isolated in embryonated eggs from Muscovy ducks in South Australia. CyRV morphologically resembles arenaviruses; however, sequencing identified CyRV as an orthomyxovirus. The high mortality rate among ducks co-infected with salmonellae suggests that CyRV may be pathogenic, either alone or in concert with other infections.

In May 2010, an outbreak of disease at a duck farm at Cygnet River on Kangaroo Island, South Australia, Australia, occurred in 4-month-old Muscovy ducks (*Cairina moschate*). The ducks had been incorrectly fed during a week-long absence of the owner from the farm. The ducks were lethargic and had diarrhea, and the mortality rate among infected ducks was high. Of 150 ducks, 128 died in a 3-day period. Despite treatment with tetracycline, only 5 of the remaining 22 ducks survived.

## The Study

After disease was discovered among Muscovy ducks on a farm in South Australia, 5 ducks with signs of infection were submitted for investigation to Gribbles Veterinary Pathology (Glenside, SA, Australia); 2 ducks died during transit. The 3 remaining ducks were euthanized, and postmortem examination showed severe necrotising fibrinous enteritis, multifocal piecemeal hepatitis, and severe fibrinous multifocal splenitis. *Salmonella enterica *serovar Typhimurium (phage type 9) was isolated from the ducks and was detected in at least 2 of the following from each animal: feces, spleen, bone marrow, and liver. Histologic lesions were consistent with a disseminated *Salmonella* infection; thus, a diagnosis of septicemic salmonellosis was made.

Pooled samples (liver, brain, lung, spleen, and gastrointestinal tissues) from 2 ducks were submitted for disease exclusion at the Australian Animal Health Laboratory, Commonwealth Scientific and Industrial Research Organization (Geelong, VIC, Australia). Results of nucleic detection assays excluded Newcastle disease virus and influenza virus as causative agents.

Isolation in 9- to 11-day-old embryonated eggs was performed on liver, brain, lung, spleen, and gastrointestinal tissues from 2 ducks after the samples were treated with antimicrobial drugs. In each of 3 passages in the embryonated eggs, embryo death occurred on passage days 4–5 for all tissues except the gastrointestinal tissues. However, when red blood cells from chicks and guinea pigs were used, hemagglutination that would indicate the presence of paramyxoviruses or influenza viruses was not observed in the allantoic fluid. In addition, the agent was filterable, suggesting the presence of a virus.

We observed hemolysis on the heads of the embryos and processed the embryos and chorioallantoic membranes for histopathologic studies. Multifocal necrosis was observed in the liver and lung of the embryos and in the chorioallantoic membranes. These necrotic lesions were consistent with an infectious agent. No influenza or Newcastle disease virus antigens were detected in the embryo and chorioallantoic membrane by immunohistochemical testing.

We observed cytopathic effects in tissues during additional passages in chicken embryo fibroblasts, Muscovy duck embryo fibroblasts, and Vero and Vero E6 cells. The cytopathic effect observed in Vero cells was minimal in comparison with that observed in other cell lines. We analyzed cultured samples from the allantoic fluid, chicken embryo fibroblasts, and Vero cells by using negative-contrast electron microscopy with nano-W stain (Nanoprobes, Yaphank, NY, USA). Vero cell monolayers were grown on sapphire disks, lightly fixed, and frozen under high pressure (Leica EM HPM100 high-pressure freezer; Leica Microsystems, North Ryde, NSW, Australia) before being freeze-substituted (*1*), infiltrated, and embedded in HM20 resin. After polymerization, the blocks were sectioned to 90 nm and counterstained with uranyl acetate and lead citrate. PCR for the causative agents of duck virus hepatitis and duck virus enteritis and for paramyxoviruses and mycoplasmae did not amplify any products.

**Figure 1:**
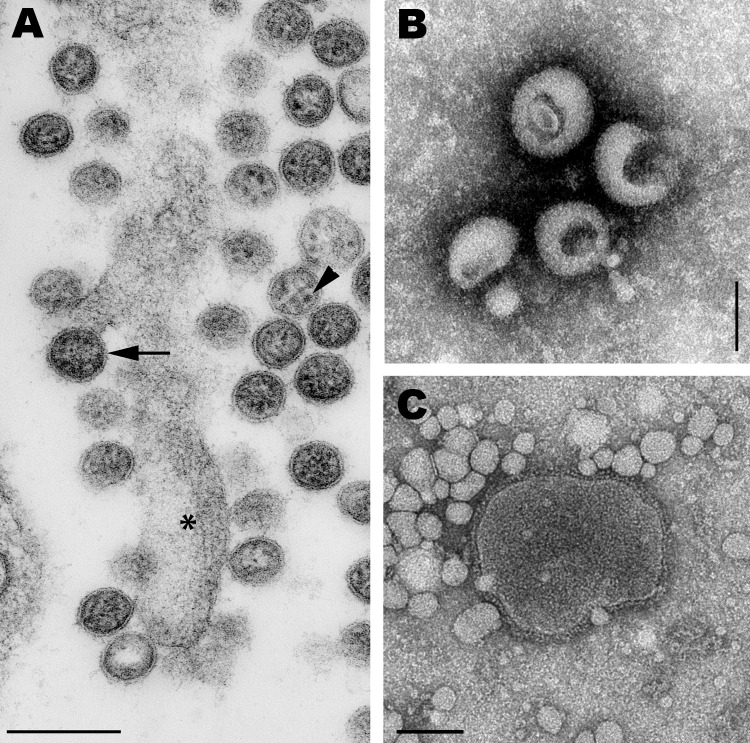
A) Transmission electron micrograph of an ultrathin section of Vero cells infected with Cygnet River virus (CyRV) from a Muscovy duck, Australia. Arrow, virus budding from the plasma membrane; arrowhead, sand-like structures. *Host cell projection. Scale bar = 200 nm. B, C) Transmission electron micrographs of CyRV prepared by negative-contrast electron microscopy. Scale bars = 100 nm. Preparations were derived from supernatant of CyRV-infected Vero cells (B) and from allantoic fluid of CyRV-infected eggs (C).

The virus grew to low titer in the allantoic fluid of eggs and in culture (in chicken embryo fibroblasts; 50% tissue culture infectious dose, 10^4.8^/mL), which made initial identification by electron microscopy difficult. Despite minimal cytopathic effect, virus propagation in cell lines was highest in Vero cells (50% tissue culture infectious dose, 10^7.9^/mL), most likely because this cell line lacks interferon. The virus particles had ultrastructural characteristics of arenaviruses and orthomyxoviruses (Figure 1).

PCR amplification using panarenavirus PCR primers (*2*) did not identify arenavirus sequences. Therefore, we performed high-throughput sequencing by using the 454 Genome Sequencer FLX system (Roche Diagnostic Australia Pty Ltd, Castle Hill, NSW, Australia) to identify the virus. We pooled the allantoic fluid and tissue culture supernatant from chicken embryo fibroblasts for clarification at 10,000 rpm (JA 25.50 rotor; Beckman Coutler, Gladesville NSW, Australia) for 20 min and then centrifugation at 36,000 rpm (SW41 rotor; Beckman Coulter) for 90 min on a 20% sucrose cushion in TNE buffer (10 mM Tris, 0.1 M NaCl, 1 mM EDTA) buffer. The virus was resuspended in 350 μL of Buffer RLT (RNeasy lysis buffer) and extracted by using the RNeasy Mini Kit (QIAGEN, Doncaster, VIC, Australia) according to manufacturer’s instructions. We performed additional processing of the sample as described (*3*). 

Sequencing resulted in 15.5 Mb of sequence, which was assembled into 1,796 contigs. We used the 30 longest contigs in a BLAST search to match sequences available in the GenBank database (*4*). No similarity was observed at the nucleotide level, so we performed a blastx (www.ncbi.nlm.nih.gov/BLAST) search. Of the 30 sequences, 3 shared sequence identity with Quaranfil virus, Johnston Atoll virus, and Lake Chad virus, respectively; these viruses are members of the proposed genus *Quarjavirus*, family *Orthomyxoviridae* (*5*). All matches were within the polymerase genes of orthomyxoviruses; Quaranfil virus was the closest match (≈60% sequence identity).

**Figure 2:**
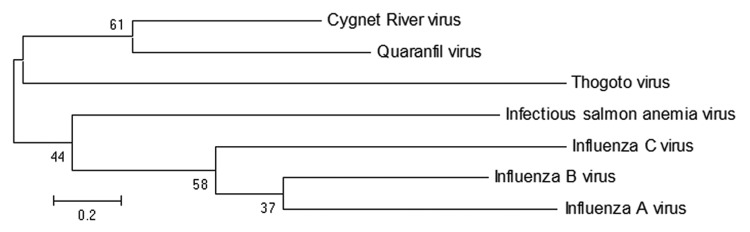
Maximum-likelihood tree showing phylogenetic relationships between Cygnet River virus isolate 10–01646 (GenBank accession no. JQ693418) and other orthomyxoviruses: Quaranfil virus isolate EG T 377 (accession no. GQ499304), Thogoto virus strain PoTi503 (accession no. AF527530), infectious salmon anemia virus isolate RPC/NB (accession no. AF435424), influenza C virus C/Yamagata/8/96 (accession no. AB064433), influenza B virus B/Wisconsin/01/2010 (accession no. CY115184), and influenza A virus A/California/07/2009(H1N1) (accession no. CY121681). Tree was based on deduced amino acid sequences of the complete matrix protein of orthomyxoviruses, applying 1,000 bootstrap replicates (*6*). Numbers at nodes indicate percentage of 1,000 bootstrap replicates. Scale bar indicates nucleotide substitutions per site.

The complete gene encoding the matrix gene was amplified by using PCR with published primers for Quaranfil virus (*5*) and then cloned and sequenced (GenBank accession no. JQ693418). The deduced amino acid sequence for the matrix protein was compared with sequences of other orthomyxoviruses and showed 31% identity with Quaranfil virus, 19% with influenza B(B/Yamagata), 18% with influenza A(A/PR/8/34), and 15% with infectious salmon anemia virus. We conducted phylogenetic analysis of the matrix protein of amino acid sequences from representative orthomyxoviruses by using the maximum likelihood method with MEGA5 (*6*) (Figure 2). Bootstrapping at 1,000 replicates was conducted. Analysis showed that this virus was novel; thus it was designated Cygnet River virus (CyRV). Further analysis is ongoing to determine the full genome sequence.

## Conclusions

We identified a novel orthomyxovirus virus isolated from Muscovy ducks in South Australia. Examination by electron microscopy showed that the virus has a strong morphologic resemblance to arenaviruses and orthomyxoviruses. Next-generation sequencing enabled identification of the virus as an orthomyxovirus (member of the family *Orthomyxoviridae*). The implications of this discovery extend to 3 areas.

First, the discovery of this novel virus will enable the development of diagnostic reagents for the future detection of the virus. The isolation of the virus also enables us to conduct in-depth pathogenesis studies (ongoing) and to assess the potential role of this virus in disease outbreaks among ducks.

Second, the discovery of this virus provided supportive evidence for the creation of a new genus within the family *Orthomyxoviridae*. The family comprises 5 known genera (*Influenzavirus A*, *B*, and *C*, *Isavirus,* and *Thogotovirus*) and 1 tentative genus (*Quarjavirus*). The proposed genus *Quarjavirus* contains the 3 virus species: Quaranfil, Johnston Atoll, and Lake Chad viruses (*5*), and, now, a fourth member—CyRV. Quaranfil virus, a human pathogen that caused a mild febrile illness in children in Egypt (*7*), and Johnston Atoll virus, a tick-borne virus of birds, were previously identified as arenaviruses on the basis of morphologic characteristics (*8*); however, on the basis of sequence identity, they were subsequently determined to be orthomyxoviruses (*5*). Quarjaviruses are tick-borne, so ticks may play a role in the transmission of CyRV.

Third, our findings highlight the value of undertaking a thorough disease investigation. To ensure that all potential causative agents are identified during an investigation, the presence of >1 agent should not be discounted. This notion was elegantly demonstrated a few years ago when Reston Ebola virus was discovered in pigs in the Philippines after an initial diagnosis of porcine reproductive and respiratory syndrome (*9*). Although the pathogenicity of CyRV infection alone in ducks remains to be investigated, the high mortality rate (97%) among ducks co-infected with salmonellae and CyRV raises the possibility that CyRV may be pathogenic, either alone or in concert with other infections. In the ducks we investigated, insufficient feeding may also have played a role in the infection dynamics and the clinical outcome.
